# The Feeding Infants and Toddlers Study (FITS) 2016: Study Design and Methods

**DOI:** 10.1093/jn/nxy035

**Published:** 2018-06-05

**Authors:** Andrea S Anater, Diane J Catellier, Burton A Levine, Karol P Krotki, Emma F Jacquier, Alison L Eldridge, Katherine E Bronstein, Lisa J Harnack, Julia M Lorenzana Peasley, Anne C Lutes

**Affiliations:** 1 RTI International, Research Triangle Park, NC; 2 Nestlé Research Center, Vers-Chez-les-Blanc, Lausanne, Switzerland; 3 Nutrition Coordinating Center, University of Minnesota, Epidemiology & Community Health, Minneapolis, MN

**Keywords:** nutritional epidemiology, Feeding Infants and Toddlers Study, FITS 2016, nutrient intakes, food intakes, eating habits, young children

## Abstract

**Background:**

Diet and feeding patterns during the infant, toddler, and preschool years affect nutrient adequacy or excess during critical developmental periods. Understanding food consumption, feeding practices, and nutrient adequacy or excess during these periods is essential to establishing appropriate recommendations aimed at instilling healthy eating behaviors in children.

**Objective:**

The objective of the 2016 Feeding Infants and Toddlers Study (FITS 2016) was to update our knowledge on the diets and feeding patterns of young children and to provide new data in related areas such as feeding behaviors, sleep, physical activity, and screen use. This article describes the study design, data collection methods, 24-h dietary recall (24-h recall) protocol, and sample characteristics of FITS 2016.

**Methods:**

FITS 2016 is a cross-sectional study of caregivers of children aged <4 y living in the 50 states and Washington, DC. Data collection occurred between June 2015 and May 2016. A recruitment interview (respondent and child characteristics, feeding practices, physical activity, screen use, and sleep habits) was completed by telephone or online. This was followed by a feeding practices questionnaire and the 24-h recall conducted by telephone. A second 24-h recall was collected for a random subsample of 25% of the total sampled population.

**Results:**

Among the 4830 recruited households with an age-eligible child, 3248 (67%) completed the 24-h recall. The respondents were more likely to be white, less likely to be Hispanic, and more highly educated than the US population of adults in households with a child <4 y of age. The sample was subsequently calibrated and weighted, and the distribution of respondents was compared with known population distributions.

**Conclusions:**

FITS 2016 provides data based on sound methods that can inform researchers, policymakers, and practitioners about the food and nutrient intakes of young children. New findings may also be compared with previous FITS studies.

## Introduction

The years before going to school, particularly the first 2 y of life, are a critical phase in the growth, development, and even future health of children (1–4). Indeed, caregiver choices shape a young child's food environment (5), which influences the development of food preferences and dietary habits (6, 7) and can in turn contribute to a child's risk of overweight and obesity (8). Moreover, early dietary patterns and preferences may persist into later life (9–11), highlighting the importance of good nutrition during the first years of a child's life. It is, therefore, imperative to develop an understanding of key factors affecting a child's diet, such as initiation and duration of breastfeeding, timing and introduction of appropriate complementary foods, and meal and snack patterns of toddlers and preschool children.

Infant feeding practices and dietary intakes, meal and snack patterns, physical activity, and other behaviors of young children have been investigated in the cross-sectional Feeding Infants and Toddlers Studies (FITS) conducted previously in 2002 and 2008 (12, 13). Overall, the FITS 2008 reported that usual nutrient intakes were adequate and similar to those in 2002. However, average intakes of toddlers and preschool-aged children exceeded the upper intake level for some nutrients, including sodium, vitamin A, and zinc. Average intakes of saturated fat in this age group were also higher than recommended, whereas average dietary fiber and potassium intakes were generally lower than the established adequate intake level. The FITS 2008 data on food consumption clearly showed that overall diet quality was lower in preschool-aged children than in infants and toddlers.

The FITS 2016 builds on the 2 previous FITS, and uses a consistent methodology to enable comparison with the earlier studies. However, the data collection instruments and study materials were enhanced in 2016 to address emerging issues in early childhood nutrition and obesity. Additional questions were included related to modifiable risk factors for obesity, such as responsive feeding (i.e., responding appropriately to baby's cues to continue or stop feeding), reasons for not breastfeeding infants, food purchasing and preparation habits, children's sleep patterns, child screen time, and household food security. The FITS datasets remain the largest food and nutrient intake data sets for children aged <4 y. The previous FITS surveys were used to inform policy and dietary interventions for toddlers (i.e., 12–24 mo). With the creation of the Birth to 24 Months Dietary Guidance Development Project (14), the FITS 2016 study has the opportunity to inform this policy initiative and continue to fill gaps in the data about dietary patterns of the youngest Americans.

The FITS 2016 is a cross-sectional survey of caregivers (i.e., parents or other guardians) of US infants, toddlers, and preschool children aged ≤4 y. The main study aims are as follows:

- Provide robust public health data about the nutrient intakes and food consumption patterns of infants, toddlers, and preschool-aged children from birth until the age of 4 y.
- Develop data to enable further research about the potential associations between demographic and lifestyle variables and the dietary patterns and nutrient intakes of children in this age group.
- Identify areas of improvement in the diets of young children and potential subpopulations who may benefit from targeted interventions.

We describe here the FITS 2016 study design and sampling plan, data collection, sample size, sample weights, and sociodemographic characteristics of the sample compared with the US population and the 2002 and 2008 FITS populations. Data analysis methods are described in the individual papers in this Supplement.

## Methods

This section describes the FITS 2016 methods, and when relevant, describes how the FITS 2016 methods compare with previous FITS approaches. The study was conducted in 3 phases: instrument development and testing, sampling frame, and data collection. These are described in the sections below. All aspects of the study related to human subjects were reviewed and approved both before and after instrument testing and associated revisions by the Institutional Review Boards of RTI International, the University of Minnesota, and the Docking Institute of Public Affairs, Fort Hays State University.

### Instrument development and testing

The instrument development and testing phase (pilot study) was conducted over 6 mo, from December 2014 to May 2015. Questionnaires were adapted from those used in FITS 2002 and 2008 to facilitate comparisons across years but at the same time permit modifications in question choices to reflect recent scientific findings on potential factors that influence diet and eating habits of US infants and young children. The full survey instrument was comprised of 4 parts: *1*) a Screener Questionnaire to identify eligible respondents; *2*) a Recruitment Questionnaire consisting of lifestyle and sociodemographic questions; *3*) a Feeding Practices Questionnaire; and *4*) one or two 24-h Dietary Recall Interviews (24-h recalls). The 2008 instrument was developed in English and translated into Spanish by a professional survey translator with experience translating for use with diverse Spanish-speaking audiences and across different dialects. For FITS 2016, the English and Spanish versions from 2008 were used as the starting point, and unchanged questions were not retranslated. **Table 1** shows the data elements collected.

**TABLE 1:** Data collection instruments and the type of data collected^1^

Data element	Recruitment Questionnaire	Feeding Practices Questionnaire	24-h Dietary Recall
Household and caregiver characteristics			
Household demographics (household composition, income, marital status)	✓		
Caregiver demographics (sex, age, race, ethnicity, education level, employment, relation to the child)	✓		
Caregiver's height and weight (reported)	✓		
Food security status^2^	✓		
WIC participation (mother and child)	✓		
SNAP participation^2^	✓		
Caregiver's perception of healthfulness of child's diet	✓		
Child characteristics			
Demographics (sex, age, race, ethnicity)	✓		
Birth weight	✓		
Food allergies and foods avoided	✓		
Medical problems that affects eating	✓		
Foods from WIC	✓		
Food acceptance/pickiness	✓		
Childcare arrangements	✓	✓	
Physical activity levels	✓		
Screen time (≥12 mo)^2^	✓		
Sleep patterns^2^	✓		
Weight and height (reported)		✓	
Developmental milestones related to feeding		✓	
Feeding practices			
Child’s weight and length at time of dietary recall		✓	
Breastfeeding		✓	✓
Reasons for stopping/never starting breastfeeding^2^		✓	
Infant formula feeding		✓	✓
Infant feeding practices (<24 mo old)	✓	✓	
Toddler feeding practices (≥24 mo old)	✓		
Family dinner eaten together	✓		
Frequency of fast food consumption	✓		
Type of water for drinking and mixing with foods	✓		✓
Use of homemade baby food^2^	✓		✓
Organic food use^2^	✓		✓
Use of pouches^2^		✓	✓
Dilution of juice with water^2^		✓	✓
Dietary recall			
Dietary supplements use			✓
Foods and beverages consumed			✓

1 Data indicate whether a data element was collected in each of the questionnaires (except the screener). A checkmark indicates it was collected, and a blank cell indicates it was not. SNAP, Supplemental Nutrition Assistance Program; WIC, Special Supplemental Nutrition Program for Women, Infants, and Children.

2 Data element added in 2016.

Considerations in modifying content from the FITS 2008 instrument included the following: *1*) the opportunity to fill gaps in knowledge of dietary behaviors during critical periods of development; *2*) evidence from empirical research showing potential associations between children's eating patterns and food preferences and health outcomes; *3*) empirical evidence showing potential relations between sociodemographic and psychosocial constructs and children's diets; and *4*) the potential to inform practitioners and public policymakers on dietary habits and feeding practices among infants, toddlers, and young children. The opinion of leading childhood nutrition experts was sought on all modifications to the questionnaires.

The FITS 2016 instrument was composed of 4 questionnaires. The Screener Questionnaire was used to establish eligibility based on the following criteria: *1*) the presence of a child aged <4 y in the household; *2*) the presence of a primary caregiver aged >18 y who was knowledgeable about the child's diet; and *3*) a willingness to participate. For households with >1 child aged <4 y, the youngest child was selected for the 24-h dietary recall.

Before administering the Recruitment Questionnaire, interviewers first obtained informed consent. The Recruitment Questionnaire included questions about household, caregiver and child demographics, factors that might influence children's eating behaviors (e.g., responsive feeding behaviors, family dinner time) and food preferences (e.g., caregiver's perception of healthfulness of child's diet). It also included specific questions about fast food consumption, childcare arrangements, physical activity, and child health (including birth weight, medical problems that affect eating, food allergies, and foods avoided) to gauge the association between breastfeeding, nutrient intake, and food choice. New questions were added to this part of the study instrument to assess the following parameters: *1*) respondent food purchasing habits [e.g., purchase of organic or Special Supplemental Nutrition Program for Women, Infants, and Children (WIC)–eligible foods] and preparation behaviors (e.g., homemade baby food); *2*) child screen time; *3*) child sleep patterns *4*) household food security; and *5*) Supplemental Nutrition Assistance Program (SNAP) participation.

The Feeding Practices Questionnaire asked about breastfeeding or formula feeding history, developmental milestones related to feeding, and reported height and weight. New questions included the following: *1*) reasons for ending breastfeeding or not breastfeeding; *2*) the use of food from pouches; and *3*) practices regarding diluting juice.

To address differences related to the introduction of foods and eating patterns between developmental stages, the questions on both the Recruitment and Feeding Practices Questionnaires varied somewhat depending on whether the child was under or over 24 mo old. For example, the Feeding Practices Questionnaire included questions about breastfeeding only for households with a child <24 mo old, and the Recruitment Questionnaire asked questions about physical activity only if the child was >24 mo old.

**FIGURE 1:**
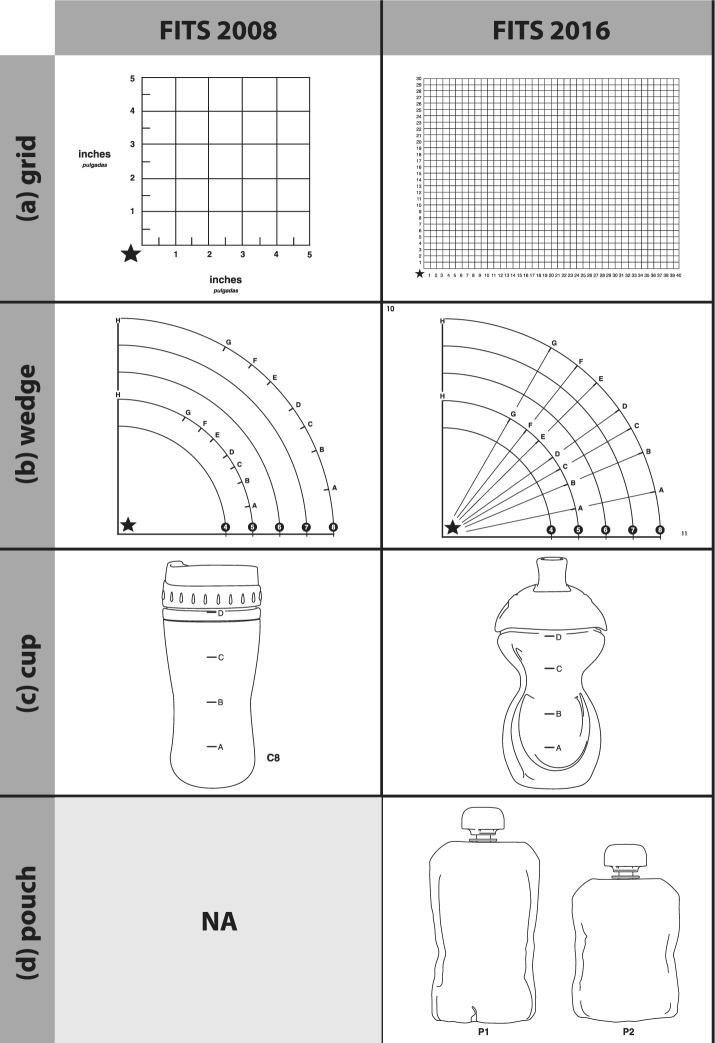
Enhancements to food measurement aids between 2008 and 2016: (A) grid; (B) wedge; (C) cup; (D) pouches.

The 24-h recall captured a detailed assessment of the selected child's diet. As in 2002 and 2008, dietary recall data were collected with the use of the Nutrient Data System for Research (NDSR). Overall, the standard interview prompts and multiple-pass approach did not change from FITS 2002 and 2008 (13). However, additional prompts were added regarding organic foods, foods eaten from a pouch, and 100% fruit juices diluted with water. Improvements and updates were made to the FITS 2008 *Food Measurement Aids* booklet sent to respondents before the 24-h recall to help them more accurately report portion sizes and to reflect market changes in shapes and sizes of cups since 2008 (**Figure 1**).

A structured instrument review process was used to evaluate new and modified questions and to ensure that the collected data effectively addressed the key research goals, but also minimized respondent burden and maximized respondents’ understanding of the questions and ability to provide accurate responses. All new and modified questions on the Recruitment and Feeding Practices Questionnaires were developed in English and translated into Spanish by a professional survey translator. A second professional translator reviewed the work of the first translator, and 2 field staff also reviewed the translation prior to cognitive testing. New and modified questions were then cognitively tested (*n* = 6) in English (with English speakers) and Spanish (with Spanish speakers). Following modifications arising from cognitive testing (e.g., addition of answer choices; rewording of new question language; clarifying instructions added for interviewers to be read as needed), the study procedures and instruments were piloted with geographically, socioeconomically, and demographically diverse representatives of our sample population, again in both English and Spanish (*n* = 17). After the pilot study and in consultation with nutrition experts, the instrument and protocol were revised (e.g., length of introduction reduced, definition of “homemade baby food” specified) and the Institutional Review Board approvals amended. Before beginning the data collection, interviewers underwent extensive training on all aspects of the questionnaires, including how to consistently address comments that were raised during the cognitive interviews or pilot.

### Sampling frame

The FITS 2016 utilized 4 sampling frames: *1*) a targeted list from a commercial vendor (Experion, Inc.) called the New Parent Database, and the Consumer Database, which was compiled with the use of proprietary methods and data sources such as parenting magazines and baby store and baby product mailing lists—we refer to this frame as the Newborn Network database; *2*) an address-based sampling frame; *3*) a targeted cellphone frame; and *4*) a web panel. FITS 2008 relied on the same targeted lists from Experion, Inc. In 2016, the efficiency of the Newborn Network database in identifying households with at least one eligible child was significantly lower than in prior surveys. Only an estimated 11% of the sampled frame members had an age-eligible child living in the household. Consequently, the yield from the list sample was much lower than in prior surveys, with <1% of sampled cases completing a 24-h recall compared with 7.3% in 2008.

To broaden coverage and increase efficiency (i.e., response rate), the Newborn Network database sample was supplemented with 2 additional sampling frames: an address-based sampling frame derived from the US Postal Service Delivery Sequence File, and a targeted cellphone frame constructed by Marketing Systems Group by appending priority data sources to the cellphone frame. These additional sampling frames resulted in 24-h recall data for only 1.4% of the address-based sample and 0.6% of the targeted cellphone sample, and so did not increase the response rate to solve the efficiency problem. To remedy this, a final frame was added, a web panel maintained by Scientific Survey International. Nearly 14% of the web panel sample provided completed 24-h recalls, with the result that half of the 24-h recall interviews came from the web panel sampling frame.

Households were selected from each frame through stratified random sampling to ensure that prespecified sample size targets by age and participation in the WIC were met. The study was designed to enable subgroup analysis within each of the age groups and to strike a balance between sampling precision and cost. Different strata were defined for the different frames to achieve sample size targets. The initial Newborn Network database frame was stratified based on child age, availability of a telephone number, and poverty quintile (as a surrogate for WIC participation). Because the goal of one-third of respondents receiving WIC was met in the Newborn Network frame without oversampling, most samples from the Newborn Network have a proportional allocation by poverty quintile and subsequent frames (address-based and cellphone) were sampled from a single stratum (households with a child in the target age range). Finally, the web sample was stratified on child age to fill in age ranges that were not yet at target sample size. As sampling progressed and the target sample sizes in different age strata were met, the age range sampled from the web panel was progressively narrowed to ensure target sample sizes in all age strata.

### Data collection

**FIGURE 2:**
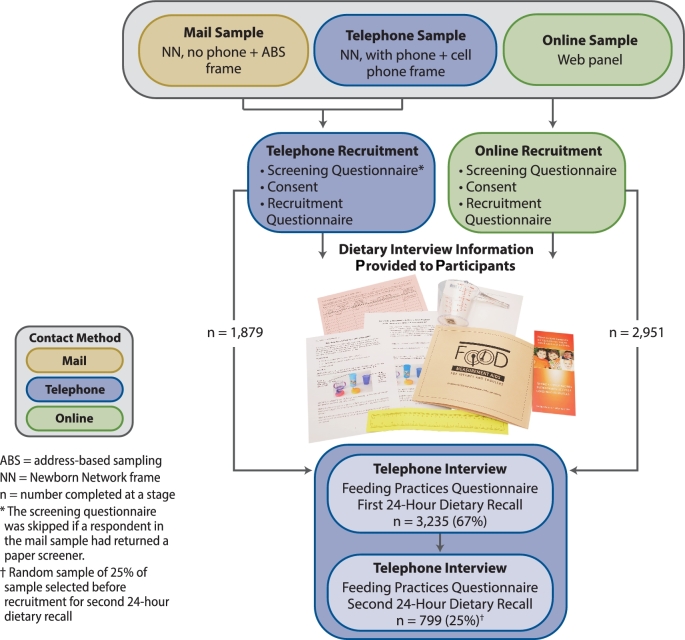
Data collection schematic for the Feeding Infants and Toddlers Study 2016.

FITS 2016 study data were collected by telephone or by the use of a combination of online and telephone, depending on the source of the sample (Figure 1). Three samples are shown in **Figure 2**: mail, telephone, and online. These designations map to the sampling frames as follows:

- **Mail sample:** The address-based frame plus about half the households from the Newborn Network frame with no telephone number (∼20% of the total subjects recruited).
- **Telephone sample:** The cellphone frame plus the other half of the households from the Newborn Network frame with a telephone number (∼20% of total subjects recruited).
- **Online sample:** The web panel frame (∼60% of total subjects recruited).

The recruitment phase consisted of screening, obtaining informed consent, and the recruitment interview. Implementation details differed among the 3 samples (mail, telephone, and online). To aid in recruitment, all respondents were offered honorariums to participate ($10 for recruitment interview, $40 for a first 24-h recall, and $25 for a second 24-h recall).

- **Mail sample:** Approximately half of the households in the mail sample completed a short screener questionnaire on paper and half of the households did this by telephone, depending on whether they were sent a paper version of the Screener Questionnaire or a postcard containing a telephone number to call to be screened and recruited. Eligible households who returned the paper screener were contacted by telephone to obtain consent and conduct the Recruitment Questionnaire. Households who telephoned to be screened and recruited were screened by telephone before obtaining consent and conducting the Recruitment Questionnaire.
- **Telephone sample:** For households with an appended telephone number, interviewers made an average of 5 call attempts. To maximize the response rate, sampled households who did not respond to multiple recruitment telephone calls were mailed letters that included information similar to what was included on the postcard for the mail sample, including a study contact number.
- **Online sample:** This sample was initially contacted by email (with information similar to the mail sample letter and postcard) and given a web address where respondents could complete the screener, provide consent, and complete the Recruitment Questionnaire.

All telephone interviews during the recruitment phase were conducted by trained interviewers at the Docking Institute with the use of a computer-aided telephone interview (CATI) system.

Following recruitment, respondents were mailed materials to assist them with accurate reporting of portion sizes for their 24-h recall. These included a ruler, a measuring cup, and a *Food Measurement Aids* booklet. All materials were in both English and Spanish. These materials were mailed by “next day” air or overnight mail (in contrast to regular mail in 2008) to improve retention between the recruitment and dietary recall phases. Respondents were asked to measure cups used by the child to drink beverages (e.g., sippy cups) per the provided instructions before the recall.

The Feeding Practices Questionnaire and the 24-h recall were completed by telephone at the same time by trained and certified dietary interviewers from the Nutrition Coordinating Center (University of Minnesota, Minneapolis, MN): the Feeding Practices Questionnaire was collected in Qualtrics, whereas the 24-h recall was conducted with the use of the NDSR 2015. In addition to facilitating collection of dietary recall data and dietary supplements consumed, NDSR also analyzes the collected data for nutrient content (consistent with FITS 2002 and 2008). The 24-h recalls were not prescheduled. Participants were asked in the recruitment interview to provide blocks of available time over a 1-wk period for the recall, and interviewers waited an average of 5 d after the dietary recall materials were mailed before contacting respondents during the time blocks. Respondents could schedule a callback time later that same day, but callbacks were not scheduled for subsequent days, to ensure that all recalls were conducted without warning. A second 24-h recall was conducted ≥1 wk later on 25% of the respondents who completed a first 24-h recall. Respondents for the second 24-h recall were selected at random during the recruitment phase. A systematic process for quality assurance (QA)/quality control (QC) was employed before, during, and after data collection, and included extensive interviewer training; monitoring of interviewers, data, and response rates during recruitment and data collection; and QC of the data following data collection.

Prior to the start of data collection for the 24-h recall, the baby foods and infant formulas in NDSR were updated to reflect the current market. The NDSR 2008 database used for FITS 2008 included 822 baby products, whereas NDSR 2015 (the version used for FITS 2016) included 1002 baby products. When respondents reported brand name products not already available in NDSR during data collection, these items were added to NDSR as User Recipes and were then available for future recalls.

Nutrients added to NDSR between the FITS 2008 and FITS 2016 data collection include total CLA; CLA *cis*-9,*trans*-11; CLA *trans*-10,*cis*-12; tagatose; vitamin D_2_ (ergocalciferol); vitamin D_3_ (cholecalciferol); added sugars (sugars and syrups added during food preparation or commercial food processing); solid fats; and 18:3 n–3 PUFA (α-linoleic acid).

FITS 2016 used 2 types of interviewers:

- **Recruitment interviewers** completed a general training session on the CATI system and a FITS-specific training session. Nearly 40 individuals completed both sets of trainings and were certified to conduct FITS recruitment calls and administer the Recruitment Questionnaire. The 2-h CATI training session provided an overview of the interviewer's role, best practices for telephone survey methods, instruction on operating the CATI system and protecting human subjects, and protocol for handling different calling dispositions (e.g., call conversions). Booster sessions were provided at the start of live data collection and then about every month or as needed. The FITS-specific training session took the interviewers through the instrument question by question, explaining the purpose of each question, defining key terms, and addressing frequently asked questions. Interviewers also conducted mock interviews using the FITS instruments.
- **Dietary recall interviewers** completed a 2-d NDSR Certification Process and a 1-d FITS Certification Process. The NDSR training consisted of developing interviewing techniques, using the multiple-pass approach, and navigating the NDSR search features. Potential interviewers also had to perform 10 practice 24-h recalls, 1 baseline recall, and 1 certification recall. The FITS training consisted of reviewing the FITS Manual of Procedures, FITS-specific data entry rules, training activities, 2 practice recalls in each of the age groups (0–23.9 mo and 24–47.9 mo), practice with administering feeding questions in Qualtrics, and certification recalls. During the FITS-specific training, additional attention was paid to special-interest items, including collecting brand names, recording the use of pouch containers and organic foods, and confirming dilution of juices.

Procedures were built into the entire study to ensure that the study protocols were followed, data collected met all acceptance criteria, and data were transferred between the recruitment and dietary recall phases in a timely manner. In addition to daily spot monitoring by call center supervisory staff, a third party (Biofortis) conducted monthly site visits to both the recruitment and dietary recall call centers to ensure that interviewers were certified and FITS protocols were being observed. In addition, the study coordinator maintained a data dashboard to track daily updates on targets, completes, WIC status, and more. Quality reviews were conducted throughout data collection to mitigate data entry errors and address issues in a timely manner. All 24-h recalls were subject to detailed quality controls pertaining to note coding and for completeness and correctness of breastfed milk calculations and unit conversions. This approach was identical to that used in FITS 2008 (13).

A random sample of 10% of total 24-h recalls collected underwent a 100% line-by-line QA review. In addition, any 24-h recalls assigned an incomplete status by the dietary recall interviewer due to missing information were also reviewed. Depending on the participants’ willingness and preferences for follow-up contact, 5–10 telephone attempts or 1 email attempt were made to contact the respondent for information related to foods consumed while the child was in a childcare setting or in the care of another adult. In cases in which the respondent could not be reached, the recall remained incomplete (*n* = 11). In cases in which the respondent could provide a list of foods consumed by the child, but could not provide the amounts consumed, the interviewers asked the respondent to provide their best estimates of usual intake for the child. If the respondent could not be reached to provide the usual intake amounts, the USDA Infant and Child Meal Patterns (15, 16) for reimbursable meals under the Child and Adult Care Food Program were used to complete the record (*n* = 23). After data collection was complete, the third-party data collection monitor also conducted a full QA on a random 10% of new user recipes created for FITS 2016 (*n* = 25).

All foods and beverages were assigned to food groups and subgroups defined for the project. The FITS 2008 food groups and subgroups were expanded to incorporate 1676 new foods and beverages added to the NDSR database since 2008 and 351 FITS-specific foods included in user recipes. Additional changes were made to reflect the current food supply, such as the addition of baby-food puffs (a grain-based first finger food), and further changes were made to align more closely with the food grouping system used in the National Health and Nutrition Examination Survey (17). Food mixtures and blends were assigned to a single major food group (e.g., baby food fruit, baby and toddler vegetables) and a single component subgroup according to the proportionately largest ingredient (apples and apple mixtures, broccoli and broccoli mixtures); however, the nutrient composition was determined from all constituent ingredients. This is consistent with the approach used in FITS 2008.

## Results

### Sample size and response rates

The numbers of completed recruitment questionnaires and 24-h recalls in each of 12 prespecified age-groups, and by WIC status subgroups, are shown in **Table 2**. The age of the child was determined at the time of the recruitment interview. Age for the 24-h recalls was determined at the time of recall. On average, the 24-h recalls occurred 1–2 wk after the recruitment interview. **Table 3** contains the sample size (i.e., the number of people attempted), the number of completed recruitment interviews, the percentage of people attempted (sample size) who completed recruitment interviews, the number of completed 24-h recalls, and the percentage of people recruited who completed the 24-h recall, by frame and contact mode.

**TABLE 2:** Completed recruitment interviews and 24-h recalls by age and WIC participation status of child^1^

		First 24-h recall		Second 24-h recall	
Age group,^2^ mo	Recruitment interview	Total, *n* (%)	WIC recipients (children), *n*	Total, *n* (%)	WIC recipients (children), *n*
0–3.9	538	305 (57)	121	84 (28)	34
4–5.9	470	295 (63)	124	65 (22)	18
6–8.9	777	468 (60)	211	104 (22)	49
9–11.9	697	434 (62)	164	91 (21)	31
12–14.9	652	412 (63)	141	107 (26)	44
15–17.9	470	308 (66)	98	70 (23)	24
18–20.9	305	251 (82)	79	59 (24)	16
21–23.9	216	162 (75)	62	43 (27)	13
24–29.9	162	144 (89)	40	36 (25)	10
30–35.9	183	161 (88)	46	47 (29)	15
36–41.9	186	159 (85)	42	53 (33)	10
42–47.9	174	136 (78)	33	40 (29)	11
Total	4830	3235 (67)	1161	799 (25)	275

1 Data are number of completed interviews. For the first 24-h recall, the percentage of the number recruited is shown in parentheses for the total completed. For the second 24-h recall, the percentage of the number completing the first 24-h recall is shown in parentheses for the total; the target was 25%. WIC, Special Supplemental Nutrition Program for Women, Infants, and Children.

2 Age group calculated independently for the recruitment interview and dietary recall interview, based on the date of completion.

**TABLE 3:** Sample size and completed interviews for the recruitment and 24-h recall by frame and contact mode^1^

			Recruitment interview		First 24-h recall		Second 24-h recall (25% sample)	
Frame	Contact mode	Sample size attempted,^2^*n*	Completed,^3^*n*	% of sample^4^	Completed,^3^*n*	% of recruited^4^	Completed,^3^*n*	% of first recall^4^
Newborn Network	Mail	69,973	698	1.0	608	87.1	180	29.4
	Telephone	90,454	742	0.8	614	82.7	164	26.6
	Telephone and mail	4533	32	0.7	29	90.6	8	27.6
ABS	Mail	13,000	202	1.6	183	90.6	50	27.3
Cellphone	Telephone	30,000	205	0.7	165	80.5	52	31.3
Web	Online	11,757	2951	25.1	1636	55.4	345	21.0
FITS 2016 total		219,717	4830	2.2%	3235	67.0	799	24.6
FITS 2008 total		46,558	4279	9.2%	3378	79.0	964	28.5

1 ABS, address based sampling; FITS, Feeding Infants and Toddlers Study.

2 Data are number of potential respondents attempted. Note that not all were eligible. The difference between number contacted and number completed includes those who did not respond to contact attempts, those who were reached but ineligible, and those who were reached and eligible but refused.

3 Data are number of people who completed the interview.

4 Data are percentage of those from previous stage that completed the interview.

### Sampling weights

Sampling weights for each frame were calculated starting with a base weight that was the inverse of the initial probability of selection (i.e., the frame count for the targeted sample divided by the sample fielded). The base weight was then adjusted for 3 factors: unknown eligibility, nonresponse, and number of eligible children in the household. The frames were then combined with the use of a compositing factor that adjusted the weights in each frame so that the average weights across frames were equal. Finally, the weights were calibrated to population totals for the following demographic characteristics: census division, age category by WIC status of child, sex of child by age category, race/ethnicity of the child by age category, and educational attainment of the caregiver.

The 2016 weighting methodology differed from the 2008 methodology in 3 ways. First, because the 2008 sample design utilized a single frame (the Newborn Network database), no compositing factor was needed to combine samples. Second, the 2008 nonresponse adjustment used weighting classes, whereas in 2016, a model-based adjustment was implemented. These first 2 differences are minor and are described for completeness. However, the third difference, different calibration methodology, has the potential to substantially affect the study estimates. In 2008, the data were calibrated to combinations of child's age category (13 levels) and the mother's race/ethnicity (4 levels). Combinations with few respondents were collapsed, resulting in a total of 44 post-stratification categories. The 2016 FITS data were calibrated to the following combinations: child's age category by child's WIC status (24 levels), child's age category by child's race/ethnicity (16 levels), child's age category by child's sex (16 levels), educational attainment of the caregiver (4 levels), and census division (9 levels).

### Sample characteristics and representativeness

Bias is the difference between the survey estimate and the true population proportion. For demographic outcomes, we have a gold standard, the US Census American Community Survey data (18), to evaluate the difference between the estimated totals and the true value. However, we are not able to estimate bias for study outcomes that lack a gold-standard comparison. If the sample matches the population closely for outcomes with known population distributions, then we have evidence that the combined effect of selection bias, coverage bias, nonresponse bias, and measurement bias is small for those outcomes, and provides some confidence that bias is small for outcomes without known population distributions. **Table 4** compares the distributions of 4 demographic variables (census division, sex of the child, race/ethnicity of the child, and educational attainment of the caregiver) for respondents in FITS 2016, 2008, and 2002 with the 2014 US population. The FITS 2016 respondents matched the US population for census division and the sex of the child rather closely. However, compared with the US population, a higher proportion of the FITS 2016 respondents were white non-Hispanic, a lower proportion were Hispanic, and a lower proportion reported low educational attainment. We assessed potential differences between the CATI and web panel respondents. The panel had fewer African-Americans than the population, balancing the excess from the CATI sample. Compared with CATI respondents, the panel respondents more closely matched the distribution of census division and marital status. The CATI respondents matched the population better for educational attainment and age category. These mismatches are common in probability surveys with telephone and mail contacts (19, 20) and likely reflect differential response by these subgroups rather than selection or coverage bias. Additionally, the weighting adjustments for nonresponse and the calibration procedure adjust for these differences.

**TABLE 4:** Comparison of 2014 US population to unweighted distributions of respondents who completed 24-h recalls in FITS 2016, 2008, and 2002^1^

Attribute	2014 population	FITS 2016	FITS 2008	FITS 2002^2^
Census division of residence				
New England	3.9	5.5	4.7	4.8
Middle Atlantic	12.2	11.8	12.4	14.5
East North Central	14.2	18.1	22.0	17.2
West North Central	7.0	8.7	12.1	9.0
South Atlantic	18.5	19.1	19.4	18.3
East South Central	5.9	6.8	7.0	6.5
West South Central	13.6	11.1	9.1	10.7
Mountain	7.8	7.6	6.7	6.9
Pacific	17.0	11.2	6.5	12.0
Sex (child)				
Female	48.8	49.1	47.2	48.8
Male	51.2	50.9	52.8	51.2
Race/ethnicity (child)				
White non-Hispanic	50.0	66.9	74.7	74.5
Black non-Hispanic	13.3	14.0	7.4	6.7
Hispanic	25.7	14.4	10.4	7.1
Other	11.0	4.6	7.5	11.7
Educational attainment (caregiver)				
Less than high school	12.6	4.3	4.2	6.3
High school	21.7	19.3	18.9	24.9
Some college	30.4	36.8	29.6	28.4
College degree or more	35.4	39.7	47.3	39.4

1 Data are percentage of total population or total completed surveys. FITS, Feeding Infant and Toddler Study.

2 FITS 2002 included infants and toddlers aged 4–23.9 mo only; FITS 2008 and 2016 include infants and toddlers aged 0–47.9 mo.

The precision of the estimates for subgroups is a function of the sample size for that subgroup. By setting specific sample size goals for each child age category and WIC participation status, the study was designed to achieve specific precision goals for these subgroups. Sample size goals were not controlled through the study design for other subgroups (e.g., African-American, Hispanic, caregiver educational attainment category, child gender). The 2 subgroups that have a lower percentage of respondents than the population are Hispanic (14.4% of respondents compared with 25.7% of the population) and educational attainment of caregiver category less than high school (4.3% of respondents compared with 12.6% of the population). There is adequate sample size to make estimates for the Hispanic subgroups, but not for the subgroup of caregivers with less than high school education.

## Discussion

FITS 2016 is the largest study in the United States to examine the dietary intake of infants and young children during a developmentally critical phase of growth and development. The findings from FITS 2016 provide researchers, educators, health professionals, caregivers, and policymakers with new data on food and nutrient intakes, feeding practices, and timing of introduction of complementary foods in population subgroups of particular interest. In addition, future analyses may wish to focus on the comparison of findings from FITS 2016 with past FITS studies, and to examine trends in the food and nutrient intakes of young children over several points in time. We expect that the FITS study results will play a central role in informing dietary guidance that targets the unique nutritional needs, eating patterns, and developmental stages of infants, toddlers, and preschool children, including new comprehensive guidance for the birth to 24-mo age group, which comprises >80% of the FITS 2016 sample.

The FITS 2016 study builds on the strong design elements of past FITS studies but also incorporates improvements and new items of data collection. In addition to the study strengths, it is important to keep in mind some study limitations when interpreting the findings. The sampling strategy was generally successful in reflecting the geographic and sociodemographic diversity of households with ≥1 child aged <4 y across the United States. The biggest difference between the population and the respondents was the distribution of educational attainment: on average, the FITS respondents were more highly educated than the population, a result that has been observed in other telephone- and address-based sample surveys. The FITS study is not a national probability sample, and we cannot determine in what ways selection bias might have affected our study sample. To remove biases due to nonresponse and coverage, we calibrated the sampling weights to the population distributions for age, sex, race/ethnicity, and educational attainment.

In addition, data were self-reported. Consequently, social desirability bias in reporting is a potential concern (e.g., individuals may have overreported consumption of healthy foods and beverages and underreported consumption of less healthy foods and beverages). Although the 24-h recall is useful for surveying dietary intake in a large group and estimating group mean intakes of diet, it does have limitations. The success of the recall depends on the memory, cooperation, and communication ability of the respondent, and requires the ability to judge portion sizes accurately. In addition, the 24-h recalls do not represent the usual diet, and respondents may under- or overreport intake; however, 24-h recalls can approximate the usual diets of populations when ≥2 recalls have been conducted on the same individual.

## Notes

Published in a supplement to *The Journal of Nutrition*. Select contents of this supplement were presented at the Experimental Biology 2017 conference in a session titled, “Informing B-24 Dietary Guidelines: Findings from the New Feeding Infants and Toddlers Study 2016,” held in Chicago, IL, April 22, 2017. Supplement Coordinators were William Dietz, Milken School of Public Health at the George Washington University and Andrea S. Anater, RTI International. The FITS research was funded by the Nestle Research Centre (Lausanne, Switzerland) through a contract with RTI International and its subcontractor, the University of Minnesota. Dr. Dietz is a compensated consultant to RTI on this project. The article contents are the responsibility of the authors and do not necessarily represent the opinions or recommendations of Nestle. Publication costs for this supplement were defrayed in part by the payment of page charges. This publication must therefore be hereby marked “advertisement” in accordance with 18 USC section 1734 solely to indicate this fact. The opinions expressed in this publication are those of the authors and are not attributable to the sponsors or the publisher, Editor, or Editorial Board of *The Journal of Nutrition*.

Supported by Nestlé Research Center, Lausanne Switzerland.

Author disclosure: EFJ and ALE are employees of the Nestlé Research Center (the funding source); ASA, DJC, BAL, KPK, KEB, LJH, JMLP, and ACL, no conflicts of interest.

Abbreviations used:

CATI: computer-aided telephone interviewing
FITS: Feeding Infants and Toddlers Study
NDSR: Nutrient Data System for Research
QA: quality assurance
QC: quality control
SNAP: Supplemental Nutrition Assistance Program
WIC: Special Supplemental Nutrition Program for Women, Infants, and Children
24-h recall: 24-h dietary recall interview

## References

[1] Taveras EM Childhood obesity risk and prevention: shining a lens on the first 1000 days. Child Obes 2016;12:159–61. 27135353 10.1089/chi.2016.0088 PMC4876524

[2] Blake-Lamb TL, Locks LM, Perkins ME, Woo Baidal JA, Cheng ER, Taveras EM Interventions for childhood obesity in the first 1,000 days: a systematic review. Am J Prev Med 2016;50:780–9. 26916260 10.1016/j.amepre.2015.11.010 PMC5207495

[3] Bhutta ZA, Das JK, Rizvi A, Gaffey MF, Walker N, Horton S, Evidence-based interventions for improvement of maternal and child nutrition: what can be done and at what cost? Lancet 2013;382:452–77. 23746776 10.1016/S0140-6736(13)60996-4

[4] Britto PR, Pérez-Escamilla R No second chances? Early critical periods in human development. Soc Sci Med 2013;97:238–40. 24084209 10.1016/j.socscimed.2013.09.001

[5] May AL, Dietz WH The Feeding Infants and Toddlers Study 2008: opportunities to assess parental, cultural, and environmental influences on dietary behaviors and obesity prevention among young children. J Am Diet Assoc 2010;110:S11–5. 21092764 10.1016/j.jada.2010.09.006

[6] Birch LL, Fisher JO Development of eating behaviors among children and adolescents. Pediatrics 1998;101(Supplement 2):539–49. 12224660

[7] Birch LL, Doub AE Learning to eat: birth to age 2 y. Am J Clin Nutr 2014;99:S723–8. 10.3945/ajcn.113.069047 24452235

[8] Pearce J, Langley-Evans SC The types of food introduced during complementary feeding and risk of childhood obesity: a systematic review. Int J Obes 2013;37:477–85. 10.1038/ijo.2013.8 23399778

[9] Yang Z, Huffman SL Nutrition in pregnancy and early childhood and associations with obesity in developing countries. Matern Child Nutr 2013;9:105–19. 23167588 10.1111/mcn.12010 PMC6860495

[10] Fiorito LM, Marini M, Mitchell DC, Smiciklas-Wright H, Birch LL Girls’ early sweetened carbonated beverage intake predicts different patterns of beverage and nutrient intake across childhood and adolescence. J Am Diet Assoc 2010;110:543–50. 20338280 10.1016/j.jada.2009.12.027

[11] Nicklaus S, Boggio V, Chabanet C, Issanchou S A prospective study of food variety seeking in childhood, adolescence and early adult life. Appetite 2005;44:289–97. 15927730 10.1016/j.appet.2005.01.006

[12] Ziegler P, Briefel R, Clausen N, Devaney B Feeding Infants and Toddlers Study (FITS): development of the FITS survey in comparison to other dietary survey methods. J Am Diet Assoc 2006;106(1 Suppl):S12–27. 16376627 10.1016/j.jada.2005.09.033

[13] Briefel RR, Kalb LM, Condon E, Deming DM, Clusen NA, Fox MK, The Feeding Infants and Toddlers Study 2008: study design and methods. J Am Diet Assoc 2010;110(12 Suppl):S16–26. 21092765 10.1016/j.jada.2010.09.005

[14] US Office of Disease Prevention and Health Promotion. Dietary guidelines: evolution 2016 [updated 9/12/2016]. Available from: https://health.gov/dietaryguidelines/evolution.asp.

[15] US Department of Agriculture (Food and Nutrition Service). Infant meal pattern 2016.

[16] US Department of Agriculture (Food and Nutrition Service). Child meal pattern 2016.

[17] US Department of Agriculture (Agricultural Research Service). What we eat in America. Beltsville, MD; 2016.

[18] US Census Bureau. American Community Survey (ACS) 2016. Available from: https://www.census.gov/programs-surveys/acs/.

[19] AAPOR Cell Phone Task Force. New considerations for survey researchers when planning and conducting RDD telephone surveys in the US with respondents reached via cell phone numbers. 2010.

[20] Kennedy C, McGeeney K, Keeter S The twilight of landline interviewing. 2016 August 1. Report No. http://www.pewresearch.org/2016/08/01/the-twilight-of-landline-interviewing/

